# Three-Dimensional Palatal Morphology and Obstructive Sleep Apnea Severity in Children with Unilateral Cleft Lip and Palate: A CBCT Study

**DOI:** 10.3390/children13070909

**Published:** 2026-07-09

**Authors:** Chinnakrij Posiri, Nuntigar Sonsuwan, Marasri Chaiworawitkul

**Affiliations:** 1 Department of Orthodontics and Pediatric Dentistry, Faculty of Dentistry, Chiang Mai University, Chiang Mai 50200, Thailand; chinnakrij_posiri@cmu.ac.th; 2 Department of Otolaryngology, Faculty of Medicine, Chiang Mai University, Chiang Mai 50200, Thailand; nuntiga.s@cmu.ac.th

**Keywords:** cleft lip and palate, sleep-disordered breathing, palatal dimensions, palatal volume, maxillary widths, cone-beam computed tomography, volumetric analysis, children

## Abstract

**Highlights:**

**What are the main findings?**

- Children with unilateral cleft lip and palate and obstructive sleep apnea showed significantly reduced palatal dimensions on three-dimensional CBCT analysis.
- Among the measured parameters, palatal volume was identified as the only variable independently associated with obstructive sleep apnea severity in this study.

**What are the implications of the main findings?**

- Reduced palatal dimensions may represent an anatomical phenotype associated with sleep-disordered breathing in children with unilateral cleft lip and palate.
- Early three-dimensional palatal assessment may support multidisciplinary cleft care and serve as a preliminary screening parameter to identify children at elevated risk for obstructive sleep apnea.

**Abstract:**

**Background/Objectives**: Obstructive sleep apnea (OSA) is highly prevalent in children with unilateral cleft lip and palate (UCLP) due to maxillary retrusion and upper airway compromise. While palatal morphology may influence pediatric OSA, three-dimensional (3D) evaluations in this population remain limited. This study evaluated palatal dimensions and maxillary widths in UCLP children with and without OSA using cone-beam computed tomography (CBCT) and examined their associations with OSA severity (apnea–hypopnea index, AHI). **Methods**: Forty CBCT scans of Thai children with non-syndromic UCLP (mean age 8.98 ± 1.99 years) were analyzed. Participants were categorized into OSA (*n* = 20; AHI ≥ 1) and non-OSA (*n* = 20; AHI < 1) groups. Reconstructed palatal structures were measured for surface area, volume, height, and transverse maxillary widths. Group differences were assessed using independent *t*-tests, and associations with AHI were examined via Pearson’s correlation and linear regression (*p* < 0.05). **Results**: Children with OSA exhibited significantly reduced palatal surface area, volume, height, and buccal alveolar crest width compared with those without OSA (*p* < 0.05). Other transverse widths showed no significant intergroup differences. Linear regression identified palatal volume as the only variable independently associated with AHI (β = −0.631, *p* < 0.001). **Conclusions**: Children with UCLP and OSA exhibit significantly constricted palatal morphology. Among the measured parameters, reduced three-dimensional palatal volume was the only variable independently associated with increased OSA severity in this sample. Thus, CBCT-based palatal volume assessment may serve as a preliminary screening parameter to help identify OSA risk within multidisciplinary cleft care, though further validation is needed.

## 1. Introduction

Obstructive sleep apnea (OSA) is a common pediatric sleep-related breathing disorder characterized by recurrent episodes of upper airway obstruction during sleep. Its prevalence in otherwise healthy children is estimated at 1% to 5%, and it has been linked to adverse neurocognitive, cardiovascular, behavioral, and growth-related outcomes [1]. Overnight polysomnography (PSG) is the gold standard for diagnosis, and the apnea–hypopnea index (AHI) derived from PSG is the most widely accepted metric for defining the presence and severity of OSA in children [2]. Adenotonsillar hypertrophy is the primary cause of pediatric OSA; however, craniofacial anomalies, particularly cleft lip and palate (CLP), are well-recognized risk factors because of their characteristic maxillary deficiency and compromised upper airway anatomy [3].

CLP arises from the disruption of the embryologic fusion of the medial nasal, lateral nasal, and maxillary processes [4], with a reported incidence of approximately 1.7 per 1000 live births in Thailand [5]. Children with CLP, especially those with unilateral cleft lip and palate (UCLP), have a significantly higher prevalence of OSA than those without CLP. Population-based data indicate that the prevalence of PSG-confirmed OSA in children with cleft lip and/or palate is approximately 6.3%, whereas no cases were identified in non-cleft controls, highlighting the markedly elevated risk in this population [6]. Structural anomalies such as midfacial retrusion, maxillary hypoplasia, and alterations in the nasal cavity, nasopharynx, oropharynx, and palate contribute to airway narrowing and increased OSA susceptibility [7]. These craniofacial deficiencies, particularly a retrusive maxilla, reduce cross-sectional airway volume and further increase the likelihood of OSA in this population [8]. Collectively, these findings demonstrate the importance of investigating specific craniofacial correlates, particularly palatal dimensions and maxillary constriction, that may underlie OSA susceptibility in children with CLP.

Recent evidence has identified palatal morphology as a potential anatomical correlate of pediatric OSA. Reduced palatal dimensions have also been found to correlate with smaller oropharyngeal airway volumes, suggesting a functional association between maxillary constriction and upper airway patency [9,10]. Furthermore, three-dimensional assessments of cleft populations have demonstrated that children with UCLP exhibit significantly smaller palatal volumes and surface areas compared with those without UCLP, findings that coincide with reduced posterior airway spaces [11]. These observations indicate that alterations in palatal morphology not only reflect craniofacial deficiency but may represent structural features associated with OSA susceptibility and severity. Importantly, because these structural constraints are potentially modifiable through early targeted orthodontic interventions, such as maxillary expansion, a detailed understanding of three-dimensional palatal configurations may aid in comprehensive treatment planning to potentially improve respiratory outcomes [1].

Although conventional assessments of palatal and maxillary morphology using two-dimensional radiographs or dental casts [12] are adequate for basic linear analysis, they offer limited insight into volumetric configuration and asymmetry [13]. Although laser scanning and digital models enhance surface visualization, they remain inadequate for defining skeletal boundaries in cleft-related deformities [14]. By contrast, cone-beam computed tomography (CBCT) enables high-resolution, three-dimensional evaluation with isotropic voxel precision and minimal geometric distortion, allowing accurate quantification of palatal surface area and volume [14,15]. This modality provides a reliable platform for examining craniofacial correlates of OSA in children with UCLP, a population in which complex skeletal asymmetries require detailed volumetric assessment.

Despite these recognized anatomical risks, comprehensive evaluations of the relationship between three-dimensional palatal volume, surface area, and OSA severity in children with UCLP remain limited. Therefore, this study quantitatively assessed palatal morphology and transverse maxillary widths in children with UCLP with and without OSA using CBCT. Associations between palatal dimensions and AHI were also analyzed to clarify the relationship between palatal configuration and OSA severity and to explore their potential relevance for clinical risk assessment.

## 2. Materials and Methods

### 2.1. Study Population and Ethical Approval

This cross-sectional study included participants who attended the Ear, Nose, and Throat (ENT) snoring clinic at the Faculty of Medicine, Chiang Mai University Hospital, as well as the Orthodontic Division, Department of Orthodontics and Pediatric Dentistry, Chiang Mai University, between 2019 and 2024. Ethical approval was granted by the Human Experimentation Committee of the Faculty of Dentistry, Chiang Mai University, Thailand (Approval No. 56/2022; approval date: 17 October 2022). Written informed consent was obtained from each participant and their caregivers prior to enrollment, permitting the use of CBCT images, polysomnographic findings, and associated medical information for research purposes.

### 2.2. Sample Size and Participants

Sample size was determined using G*Power software (version 3.1.9.4; University of Kiel, Germany) [16]. The calculation was based on an effect size of 0.96, derived from a prior study examining three-dimensional palatal morphology in patients with OSA [9], with a statistical power of 0.80 and a significance level of α = 0.05 for a two-tailed test, yielding 20 participants per group.

A total of 40 Thai children (5–12 years old) diagnosed with non-syndromic UCLP were recruited. Prior to study inclusion, all candidates underwent a comprehensive clinical assessment by an otolaryngologist. Demographic data—including age, sex, weight, and height—were recorded, and body mass index (BMI) was computed to evaluate nutritional status. During the examination, tonsillar size was graded according to the Brodsky classification [17], and adenoid size percentage was recorded for each participant. To ensure airway stability prior to data collection, participants identified with adenotonsillar hypertrophy were prescribed a standardized six-week course of medical therapy under otolaryngological supervision before proceeding with polysomnography (PSG) and CBCT imaging.

To eliminate potential anatomical and physiological confounders, strict exclusion criteria were applied. Children were excluded if they presented with extreme BMI values (below the 5th or above the 95th percentile), persistent Grade III–IV tonsillar hypertrophy following medical management, or a diagnosis of central or mixed sleep apnea. Furthermore, a history of orthodontic interventions, continuous positive airway pressure (CPAP) therapy, secondary alveolar bone grafting, or major craniofacial surgery disqualified candidates from the study, as did the presence of incidental pathological lesions on CBCT scans.

### 2.3. PSG and OSA Diagnosis

All eligible patients underwent overnight PSG using a Type III portable device, SOMNOlab-2 (Weinmann, Hamburg, Germany) in the Department of Otolaryngology, Faculty of Medicine, Chiang Mai University. PSG recordings included oronasal airflow, snoring, thoracoabdominal movements, pulse rate, and arterial oxygen saturation. The AHI was calculated as the number of apnea and hypopnea events per hour of sleep. Pediatric OSA was diagnosed when the AHI was ≥1 events/h, and cases were further classified as mild (AHI ≥ 1 to <5), moderate (AHI ≥ 5 to <10), or severe (AHI ≥ 10) [1]. Following these criteria, the sample was divided into two groups: an OSA group (AHI ≥ 1, *n* = 20) and a non-OSA group (AHI < 1, *n* = 20).

### 2.4. CBCT Assessment and Measurements

Image Acquisition and Safety Protocol. To minimize temporal variation, PSG and CBCT examinations were performed within one month of each other. Importantly, CBCT imaging was performed strictly as part of routine pre-surgical and orthodontic diagnostic planning prior to alveolar bone grafting; no additional radiation exposure was incurred solely for research purposes.

All CBCT scans were acquired using a MobiiScan unit (NECTEC, Pathumthani, Thailand) operating with a voxel resolution of 0.4 mm, a total scanning duration of 26 s, and a field of view of 16 cm × 16.8 cm [18]. During image acquisition, participants were positioned supine with the maxillary and mandibular teeth in maximum intercuspation. Strict instructions were given to avoid swallowing or head movement to prevent motion artifacts.

**Figure 1:**
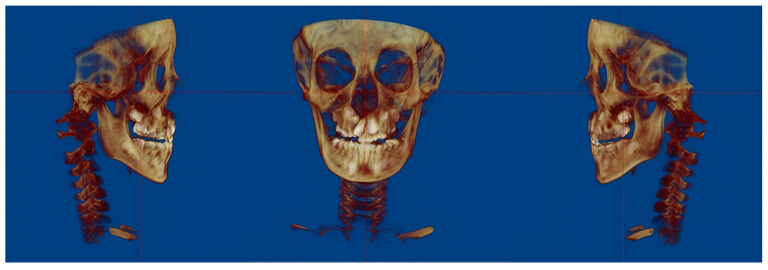
Standardization of CBCT datasets using the Frankfort horizontal plane for skull orientation in Dolphin Imaging software.

Image Orientation and Linear Measurements. CBCT datasets were imported into Dolphin Imaging software (version 11.9; Dolphin Imaging & Management Solutions, Chatsworth, CA, USA) and standardized using the Frankfort horizontal (FH) plane for skull orientation (Figure 1).

**Figure 2:**
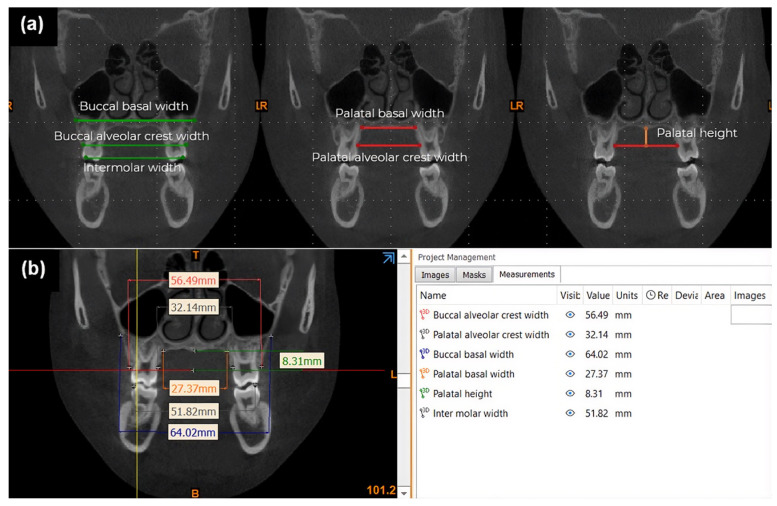
Linear measurements of palatal dimensions and transverse maxillary widths on the most anterior coronal CBCT slice at the level of the maxillary first molars in Mimics Medical software. (**a**) Illustration of measurement landmarks. (**b**) Representative coronal CBCT slice showing linear measurements used for analysis.

Linear measurements of palatal dimensions and maxillary width were performed using Mimics Medical software version 21.0 (Materialise, Leuven, Belgium). Transverse maxillary dimensions were obtained according to the method described by Podesser et al. [19], whereas vertical palatal height was measured following the protocols described by Yassaei et al. [15]. The most anterior coronal slice displaying the complete palatal roots of the maxillary first molars was selected for analysis (Table 1; Figure 2). To minimize measurement bias from dental anomalies common in UCLP (crowding, ectopic eruption, and missing teeth), transverse dimensions were defined using skeletal landmarks independent of dental position. The assessed parameters included intermolar width, buccal and palatal alveolar crest widths, and buccal and palatal basal widths. Detailed definitions and anatomical landmarks for all linear measurements are comprehensively outlined in Table 1.

**Figure 3:**
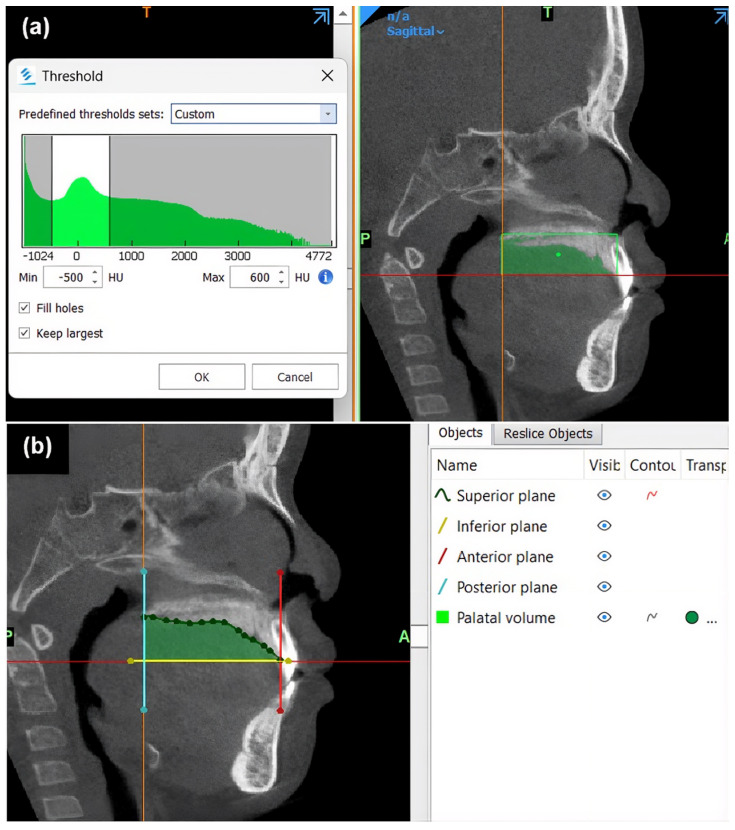
Palatal volumetric assessment in Mimics Medical software. (**a**) Threshold-based segmentation of the palatal region (indicated by the green area). (**b**) Delineation of boundary planes used for palatal volume measurement. The colored lines represent the superior (green), inferior (yellow), anterior (red), and posterior (cyan) boundary planes.

**Figure 4:**
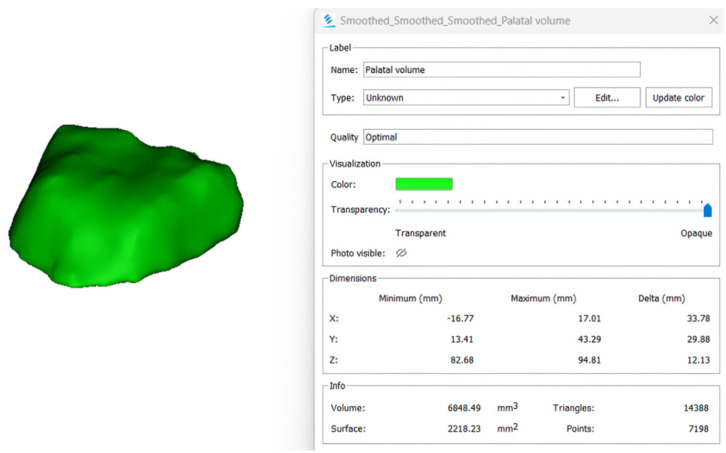
Three-dimensional reconstruction of palatal morphology following manual segmentation, used for palatal surface area and volume calculation in Mimics Medical software.

Three-Dimensional Volumetric Assessment. Volumetric assessment of the palate was based on tissue density using a Hounsfield unit (HU) threshold of −500 to 600 in Mimics Medical software on the same Digital Imaging and Communications in Medicine (DICOM) datasets (Figure 3a). The superior, inferior, anterior, posterior, and lateral boundaries of the palatal morphology were then defined according to a standardized multiplanar protocol adapted from Yu et al. [14] and Huang et al. [20]. The superior plane corresponded to the nasal floor, ensuring inclusion of the hard palate without overlap from nasal or sinus structures. The inferior plane was defined by three reference points: (1) the palatal side of the anterior alveolar crest along the mid-sagittal plane and (2–3) the midpoints of the palatal alveolar crests of the right and left first molars. The anterior and posterior boundaries were delineated by coronal planes passing through the anterior alveolar crest and the posterior nasal spine, respectively, whereas the lateral borders followed the internal contours of the palatal bone (Figure 3b). Manual delineation and slice-by-slice segmentation were performed to ensure accurate boundary definition, allowing the software to automatically calculate palatal surface area (mm^2^) and palatal volume (mm^3^) (Figure 4).

### 2.5. Reliability Analysis

To assess intra-examiner reliability, the primary investigator repeated all linear and volumetric measurements on 10 randomly selected CBCT datasets after a four-week interval. The mean of the two readings was subsequently used for the main analysis. To evaluate inter-examiner reliability, the same 10 CBCT datasets were independently analyzed by a second calibrated orthodontist. Agreement within and between examiners was quantified using the intraclass correlation coefficient (ICC).

### 2.6. Statistical Analysis

All statistical analyses were conducted using IBM SPSS Statistics version 25.0 (IBM Corp., Armonk, NY, USA). Descriptive statistics (mean ± standard deviation) were computed for continuous variables. Data normality was confirmed using the Shapiro–Wilk test. Chi-square tests were employed to evaluate differences in categorical variables between the groups, while independent samples *t*-tests were used to compare intergroup differences for continuous variables (palatal dimensions and maxillary widths). Pearson’s correlation analysis was performed to examine the associations between the studied variables and the AHI.

To identify variables independently associated with OSA severity, a multivariable linear regression model was constructed. Only variables demonstrating statistically significant univariate correlations with AHI were included in the regression model to prevent model overfitting and ensure that variables lacking significant associations were appropriately excluded. Standardized β coefficients and R^2^ values were reported. For all statistical tests, a *p*-value of <0.05 was considered statistically significant.

## 3. Results

### 3.1. Demographic and Clinical Characteristics

Table 2 summarizes the demographic data of the 40 Thai patients with UCLP (16 females, 24 males; mean age, 8.98 ± 1.99 years). Participants were classified into an OSA group (*n* = 20; mean age = 9.25 ± 1.71 years) and a non-OSA group (*n* = 20; mean age, 8.70 ± 2.25 years). The two groups demonstrated similar mean BMI values (OSA: 17.94 ± 4.27 kg/m^2^; non-OSA: 17.37 ± 2.70 kg/m^2^). No significant intergroup differences were observed in adenoid size percentage or tonsil grade distribution (*p* = 0.733 and *p* = 0.388, respectively). The mean AHI was 3.79 ± 3.30 events/h in the OSA group and 0.42 ± 0.26 events/h in the control group. Among children in the OSA group, OSA severity was classified as mild in 13 participants, moderate in 5 participants, and severe in 2 participants.

### 3.2. Measurement Reliability

According to the 95% confidence intervals, the intraclass correlation coefficients (ICCs) for intra- and inter-examiner reliability demonstrated excellent agreement across all parameters, confirming high measurement reproducibility:

- Palatal volume: 0.998 and 0.997, respectively;
- Palatal surface area: 0.995 and 0.976, respectively;
- Palatal height: 0.986 and 0.934, respectively;
- Intermolar width: 0.980 and 0.940, respectively;
- Buccal basal width: 0.984 and 0.953, respectively;
- Palatal basal width: 0.993 and 0.986, respectively;
- Buccal alveolar crest width: 0.994 and 0.978, respectively;
- Palatal alveolar crest width: 0.995 and 0.987, respectively.

### 3.3. Intergroup Comparison of Palatal and Maxillary Dimensions

All data were normally distributed (Shapiro–Wilk normality test; *p* > 0.05). The OSA group exhibited significantly smaller palatal volume, palatal surface area, and palatal height than the non-OSA group (*p* < 0.05). By contrast, none of the maxillary width parameters differed significantly between groups, except for the buccal alveolar crest width, which was significantly narrower in the OSA group (*p* < 0.05) (Table 2).

### 3.4. Correlations with OSA Severity

Pearson’s correlation analysis was performed to assess the relationships between the studied variables and the AHI (Table 3). No significant correlations were observed between AHI and the following parameters (all *p* > 0.05):

- Age (*r* = 0.064);
- Intermolar width *(r* = −0.260);
- Buccal basal width (*r* = −0.205);
- Palatal basal width (*r* = −0.302).

However, statistically significant negative correlations were identified for the remaining dimensions:

- Weak negative correlations: Palatal height (*r* = −0.380; *p* < 0.05) and palatal alveolar crest width (*r* = −0.392; *p* < 0.05);
- Moderate negative correlations: Palatal volume (*r* = −0.631; *p* < 0.001), palatal surface area (*r* = −0.619; *p* < 0.001), and buccal alveolar crest width (*r* = −0.524; *p* < 0.05).

### 3.5. Multivariable Linear Regression Analysis

Multivariable linear regression analysis identified palatal volume as the only variable independently associated with AHI (*p* < 0.001). Conversely, palatal surface area, palatal height, and both buccal and palatal alveolar crest widths showed no significant independent associations with OSA severity (Table 4).

## 4. Discussion

The present study showed that children with UCLP who had OSA exhibited significantly reduced palatal dimensions, including palatal volume, surface area, and height, compared with those without OSA. Among the various transverse and vertical parameters evaluated, multivariable linear regression analysis identified palatal volume as the sole variable independently associated with OSA severity (AHI). This finding suggests that three-dimensional palatal constriction may be associated with pediatric sleep-disordered breathing in this cohort.

To minimize potential confounders such as adenotonsillar hypertrophy, obesity, and sex, individuals with Grade III–IV tonsillar hypertrophy or a BMI above the 95th percentile were excluded. In addition, no significant intergroup differences were observed in tonsil grade distribution or adenoid size percentage, indicating comparable baseline adenotonsillar status within this cohort. Enlarged tonsils may constrict the oropharyngeal airway [21]; furthermore, in general pediatric populations, severe adenotonsillar hypertrophy and obesity are recognized as the primary clinical determinants of OSA, and their presence may obscure the respiratory contribution of craniofacial structures [2]. This methodological approach was intended to reduce confounding and improve the specificity of the observed associations between palatal dimensions and AHI, thereby reducing the likelihood that these associations were driven primarily by adenotonsillar enlargement, soft-tissue obstruction, or obesity-related factors [22]. Consequently, although BMI, tonsil grade, and adenoid size were documented to characterize the baseline status of the cohort, they were not included in the primary correlation and regression models, which focused on skeletal palatal and maxillary parameters. Consistent with this rationale, previous studies have shown that when extreme obesity is controlled, the associations between BMI and AHI become weak [23]. Furthermore, the literature confirms a lack of sex-related differences in OSA among patients with cleft palate [24], as the cleft anomaly itself exerts a fundamentally greater influence on maxillary morphology than sex-related growth patterns [25]. Findings from Silvestre et al. and Ho et al. have similarly indicated that sex-related variations in OSA risk are minimal in CLP populations [24,26]. Together, these methodological considerations allowed a focused evaluation of the association between palatal morphology and OSA severity within this cohort.

Historically, characterization of the palate and maxilla has primarily relied on two-dimensional linear and angular measurements from dental cast analysis [12]. Although this method is reliable, it is time-consuming and provides limited information on three-dimensional morphology [13]. To address these limitations, several studies have introduced three-dimensional imaging systems employing laser scanning to generate dental models, facilitating improved visualization and documentation of the upper arch in patients with clefts [10]. However, such surface-based techniques may not fully represent the intricate palatal anatomy [14]. In the present study, CBCT was used to obtain precise measurements of hard tissue structures, including the palate and maxilla. This technique supports both linear and volumetric assessments [15] and enables high-resolution three-dimensional reconstruction that accurately captures the complexity of palatal morphology [14,15]. CBCT-based evaluation provides reproducible and clinically applicable data for orthodontic diagnosis and interdisciplinary management of patients with OSA.

Previous studies have demonstrated craniofacial structural alterations in patients with OSA, particularly reduced maxillary dimensions and retrusion [27]. The association between maxillary morphology and upper airway dimensions indicates that they may play a role in OSA pathogenesis [7]. Kecik et al. reported significantly smaller palatal dimensions in individuals with OSA [9]. Children with UCLP are especially predisposed to OSA because of midfacial deficiency and maxillary retrusion, which contribute to reduced airway volume [8]. Comparative analyses have consistently shown smaller palatal dimensions in patients with UCLP than in non-cleft individuals [13,28], suggesting increased OSA susceptibility. In our analysis, the employment of skeletal landmarks to define transverse dimensions provided a more accurate representation of true maxillary width [19]. Most transverse parameters, including intermolar, palatal basal, and buccal basal widths, showed no significant differences between groups, except for the buccal alveolar crest width, which was significantly smaller in the OSA cohort. These findings are consistent with studies by Ciavarella et al. [10] and Johal and Conaghan [7], who reported no significant posterior transverse discrepancies between individuals with and without OSA, but contrast with the study by Kecik [9], who observed narrower dental arches in patients with OSA. Intercanine width was not included in the present analysis because many subjects (ages 5–12) were in early mixed dentition, where ongoing canine eruption may compromise measurement reliability. Nevertheless, AHI demonstrated significant negative correlations with palatal height, buccal alveolar crest width, and palatal alveolar crest width, whereas intermolar width showed no significant correlation. Maxillary constriction is a known risk factor for OSA [27]. Although Seto et al. suggested that palatal height alone may not reflect maxillary constriction [27], our findings, consistent with Ciavarella et al. [10], indicate that reduced palatal height correlates with greater OSA severity, possibly due to posterior and inferior tongue displacement leading to airway narrowing [29]. Beyond linear parameters, the three-dimensional analyses in the present study showed significant reductions in palatal surface area and volume among patients with OSA, consistent with the findings of Kecik [9] and Ciavarella [10], who reported strong negative correlations between palatal dimensions and OSA indices. In patients with UCLP, maxillary constriction and reduced palatal dimensions may contribute to compromised airway anatomy through altered tongue–palate spatial relationships and retroglossal airway narrowing, which may aggravate obstructive events during sleep [14,30].

The observed associations between palatal dimensions and OSA severity have practical implications for the multidisciplinary management of children with UCLP. Three-dimensional CBCT assessment of palatal volume may serve as a preliminary screening parameter to help identify children at elevated risk for OSA, who could then be considered for polysomnographic evaluation. However, it should be emphasized that while three-dimensional palatal volume may indicate a structural predisposition to upper airway compromise, it serves merely as an adjunctive screening tool and cannot substitute for definitive polysomnography. These findings align with reports by Watanabe et al. [31], who demonstrated that craniofacial skeletal deficiencies are associated with airway narrowing. Incorporating palatal dimension assessment into routine orthodontic planning may support early identification of OSA risk. Recognizing this structural susceptibility highlights the potential clinical value of early intervention. Targeted orthodontic treatments, such as maxillo-mandibular expansion, may help increase this three-dimensional anatomical volume. By widening the skeletal and nasal cavity base, these interventions have been shown to improve upper airway volume and potentially reduce airway resistance, offering beneficial effects for growing subjects with OSA [30]. Left untreated, OSA in patients with UCLP can lead to neurocognitive deficits, behavioral issues, impaired growth, and cardiovascular complications, making early identification and management essential [1,2].

This study has several strengths. Strict exclusion criteria (orthodontics, CPAP, alveolar bone grafting, central/mixed apnea, severe adenotonsillar hypertrophy, extreme BMI) minimized confounding and strengthened internal validity. Intra- and inter-examiner reliability were excellent (ICCs ≥ 0.93 for all parameters), confirming high measurement reproducibility. CBCT scans were acquired using a standardized protocol, and palatal volumetry used a validated HU threshold (−500 to 600) with manual segmentation blinded to group status. Children aged 5–12 years were intentionally selected because this period corresponds to active maxillary growth and routine pre–alveolar bone graft imaging, providing ample data. Palatal volume was the strongest independent correlate of AHI among the measured parameters, indicating an association between smaller palatal volume and increased OSA severity within this study cohort.

Despite these strengths, several limitations should be acknowledged. First, the cross-sectional design precludes causal inference; however, strict exclusion criteria minimized confounding, and palatal volume remained the strongest independent correlate of AHI among the measured parameters. Longitudinal studies are needed to establish causality. Second, the age range (5–12 years) corresponds to active maxillary growth, and transverse dimensions continue to change during this period [32]; groups were matched by age to minimize residual variation. Third, while manual slice-by-slice segmentation remains the reference standard for precise three-dimensional volumetric reconstruction, the process is highly labor-intensive, time-consuming, and inherently subject to inter- and intra-expert variability [33,34]. Fourth, the lack of detailed records on primary palatoplasty technique and timing limited our ability to assess whether surgical factors influenced maxillary growth, palatal morphology, and subsequent airway outcomes, including OSA [35,36]. Fifth, while strict exclusion criteria strengthened internal validity, results may not generalize to UCLP children with severe adenotonsillar hypertrophy or extreme BMI. Sixth, the utilization of a Type III portable sleep monitor (SOMNOlab-2), which inherently lacks electroencephalography (EEG) channels, presents a minor technical limitation. Because the device computes the AHI based on total recording time rather than true total sleep time, the severity of OSA could theoretically be underestimated if a child experienced significant periods of wakefulness during the night. However, this limitation was substantially mitigated in our study protocol, as all sleep examinations were performed during formal hospital admission under the direct supervision of clinical staff, ensuring that the total recording period closely reflected the actual sleep time. Seventh, the current investigation focused primarily on transverse skeletal widths, vertical height, and three-dimensional volumetric configuration, without incorporating independent linear measurements in the sagittal plane. Although sagittal boundaries were strictly controlled during the three-dimensional volume segmentation, the absence of standalone sagittal parameters limits a fully comprehensive multiplanar assessment.

Future studies with larger sample sizes should examine the effects of surgical technique, timing of palatal repair, velopharyngeal insufficiency (VPI) management, and tongue volume on OSA in UCLP. Furthermore, incorporating standalone sagittal parameters alongside longitudinal designs integrating 3D airway and soft tissue parameters would provide further insight into the anatomical mechanisms associated with OSA in this population. Automated or Artificial Intelligence (AI)-based segmentation methods could also reduce the labor-intensive nature of current 3D reconstruction workflows.

## 5. Conclusions

In conclusion, the findings of this study suggest that children with UCLP who have OSA exhibit a significantly constricted palatal morphology—specifically reduced palatal volume, surface area, height, and buccal alveolar crest width—compared to those without OSA. Furthermore, among the measured parameters, reduced three-dimensional palatal volume was the only variable independently associated with increased OSA severity in this specific sample (β = −0.631, *p* < 0.001). While two-dimensional measurements showed limited independent correlations, 3D volumetric analysis provided a more comprehensive understanding of the skeletal structural parameters evaluated. Incorporating CBCT-based palatal volume assessment into routine pre-surgical and orthodontic evaluations may serve as a preliminary screening parameter to help identify children with UCLP who are at elevated risk for OSA. Identifying this structural predisposition offers potential clinical utility by providing a rationale for early orthodontic interventions to expand palatal volume. However, further validation is needed before widespread clinical implementation. Ultimately, because these associations reflect only the hard-tissue dimensions evaluated in this sample, timely polysomnographic evaluation and comprehensive multidisciplinary management remain essential.

## Figures and Tables

**Table 1:** Definitions of palatal and maxillary measurements used in this study.

Category	Measurement Variable	Definition
Palatal dimensions	Palatal volume (mm^3^)	The three-dimensional volume of the palate.
	Palatal surface area (mm^2^)	The three-dimensional surface area of the palate.
	Palatal height (mm)	The perpendicular distance from the plane connecting the mid-palatal cemento-enamel junctions of the right and left first molars to the median palatal line.
Maxillary width	Intermolar width (mm)	The linear distance between the mesiobuccal cusp tips of the right and left first molars.
	Buccal basal width (mm)	The linear distance between the point where the most vertical line of the alveolar process transitions to the nearly horizontal plane of the lower border of the zygomatic bone process
	Palatal basal width (mm)	The linear distance between the point representing the outermost points of the palatal base
	Buccal alveolar crest width (mm)	The linear distance between the most occlusal points of the buccal alveolar process on the first molar coronal slice.
	Palatal alveolar crest width (mm)	The linear distance between the most occlusal points of the palatal alveolar process on the first molar coronal slice.

Definitions of transverse maxillary measurements were adapted from Podesser et al. [19], vertical palatal height from Yassaei et al. [15], and palatal volumetric parameters from Yu et al. [14] and Huang et al. [20].

**Table 2:** Descriptive statistics and group comparisons of subject characteristics, polysomnographic findings, and palatal and maxillary measurements.

Variable		UCLP Without OSA (*n* = 20)		UCLP with OSA (*n* = 20)		*p*-Value
		Mean ± SD	*N*	Mean ± SD	*N*	
Age (years)		8.70 ± 2.25		9.25 ± 1.71		0.390 ^a^
Sex						
	Male		11 (55%)		13 (65%)	0.519 ^b^
	Female		9 (45%)		7 (35%)	
BMI (kg/m^2^)		17.37 ± 2.70		17.94 ± 4.27		0.617 ^a^
Adenoid (%)		52.5 ± 26.92		54.95 ± 16.96		0.733 ^a^
Tonsil grade						
	0	4		7		0.388 ^b^
	I	10		6		
	II	6		7		
Polysomnographic findings						
AHI (events/hr)		0.42 ± 0.26		3.79 ± 3.30		<0.001 **^a^
Severity of OSA						
	Mild (AHI ≥ 1 to <5)			13		
	Moderate (AHI ≥ 5 to <10)			5		
	Severe (AHI ≥ 10)			2		
Palatal dimensions	Palatal volume (mm^3^)	9259.74 ± 1717.24		6944.93 ± 2086.34		<0.001 **^a^
	Palatal surface area (mm^2^)	3172.29 ± 400.21		2532.64 ± 483.87		<0.001 **^a^
	Palatal height (mm)	11.14 ± 2.19		9.82 ± 1.75		0.042 *^a^
Maxillary widths	Intermolar width (mm)	54.50 ± 3.89		53.41 ± 3.86		0.376 ^a^
	Buccal basal width (mm)	64.18 ± 3.62		63.26 ± 3.02		0.387 ^a^
	Palatal basal width (mm)	27.58 ± 3.40		25.66 ± 4.95		0.161 ^a^
	Buccal alveolar crest width (mm)	57.88 ± 3.66		55.27 ± 4.07		0.039 *^a^
	Palatal alveolar crest width (mm)	34.61 ± 2.47		33.18 ± 5.66		0.311 ^a^

UCLP = unilateral cleft lip and palate; OSA = obstructive sleep apnea; BMI = body mass index; kg = kilogram; m = meter; AHI = Apnea-Hypopnea Index; hr = hour. ^a^ Independent samples *t*-test. ^b^ Chi-square test. * Statistically significant at *p* < 0.05. ** Statistically significant at *p* < 0.001.

**Table 3:** Pearson’s correlations between AHI and study variables.

Variable	Pearson’s Correlation Coefficient (*r*)	*p*-Value
Age (years)	0.064	0.697
Palatal volume (mm^3^)	−0.631	<0.001 **
Palatal surface area (mm^2^)	−0.619	<0.001 **
Palatal height (mm)	−0.380	0.016 *
Intermolar width (mm)	−0.260	0.105
Buccal basal width (mm)	−0.205	0.205
Palatal basal width (mm)	−0.302	0.058
Buccal alveolar crest width (mm)	−0.524	0.001 *
Palatal alveolar crest width (mm)	−0.392	0.012 *

* Statistically significant at *p* < 0.05. ** Statistically significant at *p* < 0.001.

**Table 4:** Multivariable linear regression analysis of AHI as the dependent variable (five covariates).

Model	Unstandardized Coefficients		Standardized Coefficients	*t*-Test	*p*-Value
	B	Std. Error	Beta		
(Intercept)	8.724	1.367	—	6.383	<0.001
Palatal volume (mm^3^)	−0.001	0.000	−0.631	−5.017	<0.001 **
Palatal surface area (mm^2^)	−0.255	0.412	−0.255	−0.830	0.412
Palatal height (mm)	−0.166	0.228	−0.166	−1.226	0.228
Buccal alveolar crest width (mm)	−0.280	0.052	−0.280	−2.010	0.052
Palatal alveolar crest width (mm)	−1.740	0.205	−0.174	−1.291	0.205

** Statistically significant at *p* < 0.001.

## Data Availability

The data presented in this study are available on request from the corresponding author. The data are not publicly available due to privacy and ethical reasons.

## Abbreviations

The following abbreviations are used in this manuscript:

2D	Two-Dimensional
3D	Three-Dimensional
AHI	Apnea–Hypopnea Index
AI	Artificial Intelligence
BMI	Body Mass Index
CBCT	Cone-Beam Computed Tomography
CLP	Cleft Lip and Palate
CPAP	Continuous Positive Airway Pressure
DICOM	Digital Imaging and Communications in Medicine
ENT	Ear, Nose, and Throat
FH	Frankfort Horizontal
HU	Hounsfield Unit
ICC	Intraclass Correlation Coefficient
OSA	Obstructive Sleep Apnea
PSG	Polysomnography
UCLP	Unilateral Cleft Lip and Palate
VPI	Velopharyngeal Insufficiency
